# Targeted therapies for advanced non-small cell lung cancer

**DOI:** 10.18632/oncotarget.26428

**Published:** 2018-12-25

**Authors:** Xiaojuan Ai, Xialing Guo, Jun Wang, Andreea L. Stancu, Patrick M.N. Joslin, Dianzheng Zhang, Shudong Zhu

**Affiliations:** ^1^ National Key Discipline of Genetics, School of Life Sciences, Central South University, Changsha, China; ^2^ Argus Pharmaceuticals, Changsha, China; ^3^ Department of Dermatology, Brigham and Women's Hospital, Harvard Medical School, Boston, MA, USA; ^4^ Division of Hematology/Oncology, Beth Israel Deaconess Medical Center, Harvard Medical School, Boston, MA, USA; ^5^ Department of Bio-Medical Sciences, Philadelphia College of Osteopathic Medicine, Philadelphia, PA, USA

**Keywords:** NSCLC, molecular target, mTOR, EGFR, PI3K

## Abstract

Lung cancer is a serious health problem and the leading cause of cancer death worldwide, due to its high incidence and mortality. 85% of lung cancers are represented by the non-small cell lung cancer (NSCLC). Traditional chemotherapy has been the main treatment option in NSCLC. However, it is often associated with limited efficacy and overall poor patient survival. In recent years, molecular targeting has achieved great progress in therapeutic treatment of cancer and plays a crucial role in the current clinical treatment of NSCLC, due to enhanced efficacy on cancer tissues and reduced toxicity for normal tissues. In this review, we summarize the current targeting treatment of NSCLC, including inhibition of the epidermal growth factor receptor (EGFR), phosphatidylinositol 3-kinase (PI3Ks), mechanistic target of rapamycin (mTOR), epidermal growth factor receptor 2 (ErbB2), vascular epidermal growth factor receptor (VEGFR), kirsten human rat sarcoma protein (KRAS), mesenchymal-epithelial transition factor or hepatocyte growth factor receptor (c-MET), anaplastic lymphoma kinase (ALK), v-Raf murine sarcoma viral oncogene homolog B (BRAF). This article may serve as a guide to clinicians and researchers alike by assisting in making therapeutic decisions. Challenges of acquired drug resistance targeted therapy and imminent newer treatment modalities against NSCLC are also discussed.

## INTRODUCTION

**Figure 1:**
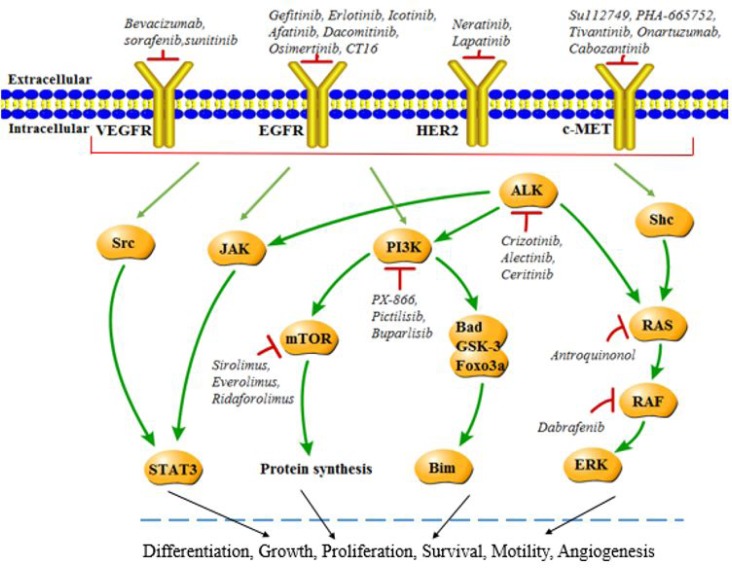
Molecular targets in NSCLC Various mechanisms including amplification and mutation may lead to activation of EGFR, PI3K, mTOR, HER2, KRAS, c-MET, ALK, BRAF and corresponding signaling pathways, while targeting inhibitors suppress the activation in NSCLC and result in therapeutic effects.

**Figure 2:**
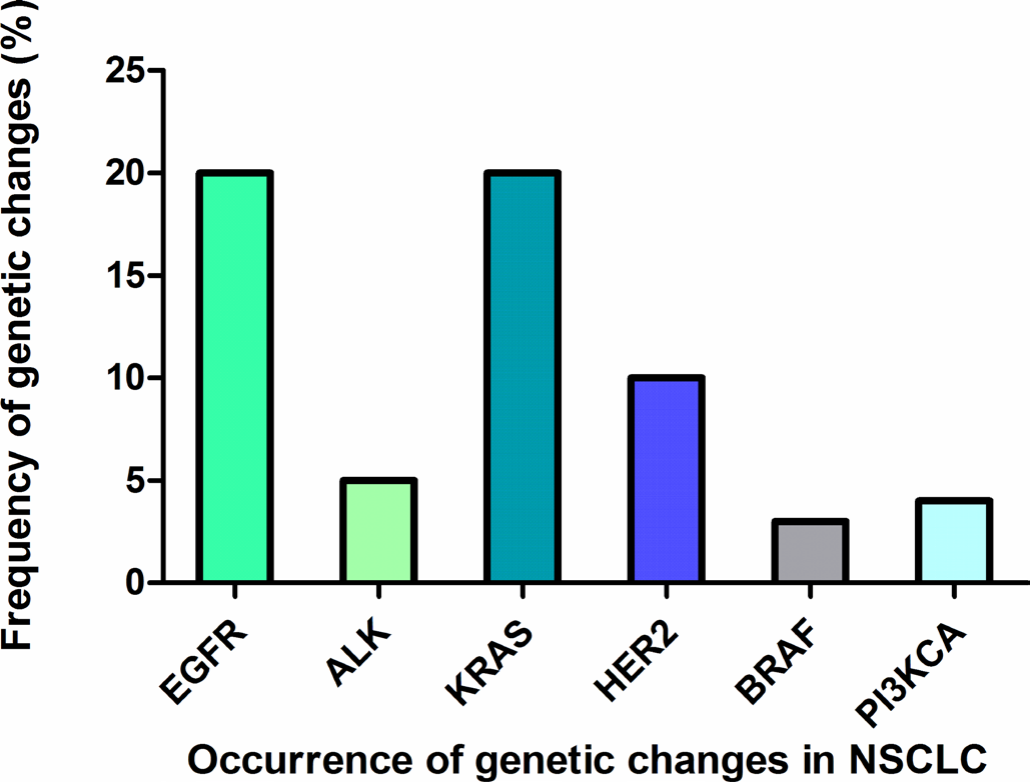
Occurrence of genetic changes in NSCLC The frequency of different genetic changes occurred in NSCLC including EGFR, ALK, KRAS, HER2, BRAF, PI3KCA.

Latest reports showed that lung cancer remains the most common fatal malignancy among males in the world [1]. Approximately 80% of cases are non-small cell lung cancer (NSCLC), and majority of NSCLC patients present with symptoms in a late advanced stage [2]. Despite recent advances in chemotherapy, the outcome for individuals with NSCLC is extremely poor. Generally, 5-year survival rate for lung cancer does not exceed 15% [3]. Thus, novel treatment strategies based on targeted therapy and targeted compounds started to be developed and implemented by researchers and clinicians. Up to now, several anti-cancer drugs targeting epidermal growth factor receptor (EGFR), phosphatidylinositol 3-kinase (PI3Ks), mechanistic target of rapamycin (mTOR), epidermal growth factor receptor 2 (ErbB2), vascular epidermal growth factor receptor (VEGFR), kirsten human rat sarcoma protein (KRAS), mesenchymal-epithelial transition factor or hepatocyte growth factor receptor (c-MET), anaplastic lymphoma kinase (ALK), v-Raf murine sarcoma viral oncogene homolog B (BRAF) have been developed, with undergoing clinical trials (Figure 1). Genetic changes of other genes have been identified (Figure 2), but EGFR, ALK and KRAS mutations are the most frequent mutations in lung cancer. To some extent, most of these inhibitors have shown promising anti-tumor efficacy, causing a paradigm shift for the treatment of advanced NSCLC, although some serious side effects, such as nausea, vomiting, hair loss and fatigue have been found in clinical trials [4–6]. In this review, we outline the recent progresses along with the emerging obstacles in NSCLC therapy.

*PFS* (Progression-free Survival): the length of time during and after the treatment of a disease, such as cancer, that a patient lives with the disease but it does not get worse *OS* (Overall Survival): the time from randomization to the date of death or the date of termination of the trial (for patients alive at the time end of the study), or the date of the last follow-up information available (for patients loss before the trial end date) *ORR* (Objective Response Rate): the number of complete plus partial response divided by the total of patients enrolled in each comparison arm.

## CHEMOTHERAPY

### Cisplatin

Cisplatin, as a non-specific drug working on cell cycle, is one of the common chemotherapeutic drugs in clinic, and acts as an anti-tumor drug mainly through suppressing DNA replication [7]. Additionally, cisplatin has anticancer activity by damaging the cellular membrane [8]. In advanced NSCLC, patients receiving cisplatin-based chemotherapy had shown significantly improved survival and quality of life along with many side-effects [9–11]. In a multinational, multicenter, open-label, phase III trial, 1125 patients with advanced NSCLC were randomly assigned to receive cisplatin alone and cisplatin plus cetuximab (an EGFR tyrosine kinase inhibitor). The results showed that patients survived longer in cisplatin plus cetuximab group than those in the cisplatin-alone group (median survival is 11.3 months versus 10.1 months; hazard ratio (HR) for death: 0.87, *p* = 0.04). Grade 3 or 4 side effects (acne-like rash:10%; diarrhea: 5%; infusion-related: 4%) were seen more in cisplatin plus cetuximab group [12]. In another phase II study, 39 patients with advanced NSCLC were randomly assigned to receive pemetrexed (a DNA/RNA synthesis inhibitor)-cisplatin (500 mg/m^2^, intravenously) concurrent with radiotherapy. The progression free survival (PFS) was 11.8 months, median overall survival (OS) was 30.3 months and time to progressive disease was 13.7 months. The response rate was approximately 46.0%. Grade 3 to 4 side effects (hematologic and esophagitis) were observed in this study [13]. These observations suggest that although cisplatin and cisplatin-based combinations have significant anticancer effects in NSCLC treatment, many side effects are also observed in the same time.

### Paclitaxel

Paclitaxel, a novel anti-microtubule drug, functions as anti-cancer reagent through maintaining tubulin stabilization and inhibiting cell mitosis. Although the clinical study showed paclitaxel’s effect on ovarian cancers and breast cancers [14, 15], it has certain curative effect on lung cancer, colorectal cancer, melanoma, head and neck cancer, lymphoma, brain tumors as well [16]. In a phase Ш trail, 134 patients with advanced NSCLC were randomly assigned to two groups: paclitaxel at 15 mg/m^2^ (three times/week for 6 weeks, *n* = 74) and paclitaxel at 45 mg/m^2^ (weekly for 6 weeks, *n* = 60). Interestingly, the response rate for low-dose paclitaxel was significantly higher than high-dose paclitaxel (83.1% vs 54.2%, *p* = 0.001). Recurrence-free survival (RFS) in low-dose paclitaxel group was also superior than in high-dose paclitaxel group (14.6 months vs 9.4 months, HR = 1.87, 95% CI (confidence interval) = 1.20–2.90, *p* = 0.005). Serious toxicities including grade 3 and 4 leukopenia/neutropenia were less occurred in low-dose paclitaxel group (*p* < 0.001) [17]. Nevertheless, a large number of side effects, such as allergic reactions, myelosuppression, neurotoxicity, cardiovascular toxicity and gastrointestinal reactions, were observed in these paclitaxel clinical trials [18–20]. Herein, a growing number of clinical trials are now investigating if nanoparticle paclitaxel could be a better treatment option as an anti-NSCLC therapy, with lower side effects. In a randomized and placebo-controlled clinical study, 92 patients with advanced NSCLC, after first-line platinum based chemotherapy failure, were randomly assigned to receive nanoparticle albumin-bound paclitaxel (nab-paclitaxel) or placebo. The median PFS was longer in nab-paclitaxel than in placebo (4.6 months vs 2.0 months; HR = 0.62, 95% CI = 0.33–0.81, *p* < 0.001). The median OS was 6.3 months versus 4.9 months in nab-paclitaxel and placebo, respectively (HR = 0.71; 95% CI = 0.33–0.85, *p* < 0.001). However, adverse events were also observed in nab-paclitaxel [21]. Similar to cisplatin and paclitaxel, nab-paclitaxel has anti-tumor efficacy with serious side effects [21].

## TARGETED SMALL-MOLECULE INHIBITORS

### EGFR mutation and EGFR tyrosine kinase inhibitors

#### EGFR mutation

Epidermal Growth Factor Receptor (EGFR, HER1) belongs to a family of ErbB receptors that includes ErbB-1, ErbB-2, ErbB-3 and ErbB-4, and is critical for tumor development, metabolic and physiological processes [22]. EGFR mutations are most prevalent and well characterized in NSCLC [23]. Numerous studies have reported that EGFR mutations frequently occurred especially in substitution of arginine for leucine at amino acid position 858 (L858R) within exon 21, and an in-frame deletion within exon 19 (delE746-A750) in NSCLC patients, which accounts for 85% of EGFR mutations [24–26]. NSCLC patients harboring EGFR^L858R^ mutation have benefited from EGFR TKIs treatments [27–30]. However, most NSCLC patients initially responding to gefitinib and erlotinib eventually become resistance [31]. In some cases, the drug resistance is a result of a second point mutation in the TK domain, in which the point mutation threonine-to-methionine substitution at position 790 (T790M) occurs in approximately one half of these acquired resistance cases [32].

### EGFR-TKI

#### Gefitinib

Gefitinib, the first generation of EGFR TKIs, was approved by Food and Drug Administration (FDA) in 2003 as the second line drug for the treatment of patients with advanced NSCLC after failure of both platinum-based and docetaxel therapies [33]. It has been newly approved by FDA as the first-line treatment of patients with metastatic NSCLC bearing EGFR exon 19 deletions or exon 21 (L858R) mutations in 2015 [33]. The post-hoc analyses of the phase III, randomized, multicenter, Iressa Pan-ASia Study (IPASS) study of 261 Asian patients (non-smokers) with EGFR mutation-positive advanced NSCLC were randomly assigned to receive gefitinib (250 mg/day) versus carboplatin/paclitaxel [34]. The median PFS suggested a statistically significant improvement with gefitinib (10.9 months) versus carboplatin/paclitaxel (7.4 months; HR = 0.54, 95% CI = 0.38–0.79, *p* = 0.0012). The objective response rate (ORR) was significantly higher with gefitinib (67.0%) than with carboplatin/paclitaxel (40.8%) (OR = 3.00; 95% CI = 1.63–5.54; *p* < 0.001). The median disease progression (DoR) was 9.6 months (95% CI = 7.4–12.5) and 5.5 months (95% CI = 4.1–5.7) in the gefitinib group and carboplatin/paclitaxel group, respectively. Nevertheless, vomiting, nausea, diarrhea and interstitial lung disease have been observed in gefitinib group [34]. EGFR inhibition and/or drug off-targets effects might cause these adverse events [35]. Thus, to minimize the side effects, identification the off-targets of gefitinib may be an important task. Recently, by the systems biology based on *silico* analysis, the crystal structure of EGFR-gefitinib complex was used to identify off-targets of gefitinib through binding pocket similarity searches on a druggable proteome database (Sc-PDB) [36]. Besides EGFR, mitogen activated protein kinase 10 (MAPK10), polymer of intrinsic microporosity-1 (PIM-1), dihydroorotate dehydrogenase (DHODH), epidermal growth factor receptor 4 (ERBB-4), 17β Hydroxysteroid dehydrogenase type 1 (HSD17B1), checkpoint kinase-2 (CHK2) as well as checkpoint kinase-1 (CHK1) also efficiently binds gefitinib [36]. Although gefitinib has shown high efficacy in patients with advanced NSCLC, drug resistance is commonly acquired when associating with EGFR T790M. Gefitinib in combination with AT-101 (a pan-Bcl-2 inhibitor) significantly inhibits cell proliferation, as well as promotes apoptosis of NSCLC cells involving blocking of B-cell lymphoma-2 (Bcl-2), B-cell lymphoma-xl (Bcl-xl), and myeloid cell leukemia-1 (Mcl-1) and downregulating of EGFR signaling [37, 38]. Animal studies further supported that the combination of gefitinib with AT-101 reinforces efficacy in NSCLC with EGFR T790M mutations [38].

#### Erlotinib

Erlotinib is another first-generation TKI that reversibly binds to EGFR approved by FDA in 2004. In 2013, it was approved by FDA as the first-line treatment for patients with metastatic NSCLC harboring EGFR exon 19 deletions or exon 21 (L858R) mutations [39]. In an open-label, randomized, multicenter, phase II study, 154 patients with advanced NSCLC with activating EGFR mutations randomly received erlotinib (150 mg/day) monotherapy or erlotinib (150 mg/day) plus bevacizumab (150 mg/kg) as a first-line therapy. The median PFS was 16.0 months in the erlotinib plus bevacizumab group (95% CI = 13.9–18.1) and 9.7 months in the erlotinib alone group (HR = 0.54, 95% CI = 0.36–0.79, *p* = 0.0015) [40]. The most common grade 3 or worse adverse events were occurred with higher frequently in the erlotinib plus bevacizumab group than erlotinib alone group (25% vs 19%). Frequency of serious adverse events (24% vs 25%) in both groups was similar [40].

#### Icotinib

Icotinib, an oral selective EGFR TKI, has been approved by the China Food and Drug Administration (CFDA). In a phase III non-inferiority trial in 27 hospitals in China, 400 advanced NSCLC patients not responding to platinum-based chemotherapy were treated with either gefitinib (250 mg/day) or icotinib (125 mg/tid). The median PFS was 4.6 months versus 3.4 months in icotinib group and gefitinib group, respectively (HR = 0.8, 95% CI = 0.7–1.1, *p* = 0.13), indicating that icotinib was non-inferior to gefitinib [41]. Drug-related adverse events, especially drug-related diarrhea (19% vs 28%, *p* = 0.033), were less occurring in icotinib group, compared with patients receiving gefitinib (61% vs 70%, *p* = 0.046) [41]. Another study has also demonstrated higher efficacy and safety with icotinib for patients with advanced NSCLC after chemotherapy treatment. In this single-arm, multi-center, prospective study, 128 NSCLC patients after chemotherapy treatment were randomly selected to receive icotinib (375 mg/day). The median PFS was 5.0 months (95% CI = 2.9–6.6) and time to progression was 5.4 months (95% CI = 3.1–7.9). The disease control rate and ORR were 67.7% and 25.8%, respectively. Rash (26%), diarrhea (12.6%) were seen in the icotinib treatment [42].

#### Afatinib

Afatinib is a second-generation EGFR TKI. It was approved by FDA in 2013 as a first-line treatment of patients with metastatic NSCLC bearing mutant EGFR (exon 19 deletions or exon 21 mutations) [43]. In randomized phase Ш trial based on 36 centers covering China, South Korea and Thailand, 910 Asian patients with advanced NSCLC were randomly assigned in three groups: afatinib (40 mg/day), gemcitabine (1000 mg/m^2^) and cisplatin (75 mg/m^2^). There was a significantly longer median PFS in afatinib group (11 months, 95% CI = 9.7–13.7) than that in either gemcitabine group or cisplatin group [44]. In another phase III study, 345 patients with stage IIIB/IV lung adenocarcinoma of EGFR mutations (exon 19 deletion, L858R, or other) were randomly assigned to either afatinib (40 mg/day) or cisplatin plus pemetrexed. It was found that the median PFS of afatinib-treated patients harboring exon 19 deletions and *EGFR* ^*L858R*^ mutation was 13.6 months, much longer than the median PFS of cisplatin plus pemetrexed group (6.9 months, HR = 0.47, 95% CI = 0.34–0.65, *p* = 0.001) [45]. Diarrhea, rash/acne, and stomatitis were observed in afatinib, while nausea, fatigue, and decreased appetite were occurred in chemotherapy [45]. Additionally, afatinib as an effective first-line treatment drug has presented definite good results in advanced NSCLC patients with EGFR-mutations who had received erlotinib, gefitinib, or both before [46–48].

#### Dacomitinib

Dacomitinib is a second-generation, irreversible TKI of EGFR that shows efficacy in NSCLC patients both in initial treatment and after failing treatment of first-generation inhibitors. The superiority of dacomitinib to its first generation TKI counterparts in advanced NSCLC was further characterized in a phase II trial: 66 patients with advanced NSCLC after failure of prior chemotherapy and erlotinib were randomly assigned to dacomitinib (45 mg/day). The median PFS was 12 weeks overall (*n* = 66) and 18 weeks (*n* = 26) for patients with EGFR mutation-positive tumors. Diarrhea, acneiform dermatitis, exfoliative rash, fatigue and stomatitis were observed in dacomitinib treatment. The results showed dacomitinib is preliminary activity and acceptable tolerability in heavily pretreated patients [49]. Clinical data showed dacomitinib has significant efficacy in advanced NSCLC patients with EGFR exon 19 deletion [50]. In the pooled subset analyses, two clinical trials were used to test the efficacy of dacomitinib in NSCLC. The ARCHER 1009 (NCT01360554) and A7471028 (NCT00769067) studies randomly assigned advanced NSCLC patients to either dacomitinib or erlotinib, while EGFR alteration examination was performed centrally on archived tumor samples. The median PFS was 14.6 months with dacomitinib and 9.6 months with erlotinib (HR = 0.717, 95% CI = 0.458–1.124; *p* = 0.146). The median survival was 26.6 months with dacomitinib versus 23.2 months with erlotinib (HR = 0.737, 95% CI = 0.43–1.259, *p* = 0.265) [50]. The results showed that dacomitinib has similar or better efficacy to erlotinib in patients with EGFR mutations. Interestingly, individuals with exon 19 deletion have more favorable outcomes with dacomitinib than with erlotinib.

#### Osimertinib (AZD9291)

Osimertinib is a third-generation TKI that binds to EGFR and was approved by FDA in 2015 for patients with metastatic *EGFR*^*T790M*^ NSCLC progressing on or after EGFR TKI therapy. NSCLC patients harboring an EGFR-activating mutation develop acquired resistance after a median of 9–11 months from the beginning of treatment with TKI, such as erlotinib and gefitinib [51, 52]. Osimertinib is a highly selective EGFR mutant inhibitor, which has been developed to be potent against EGFR mutations, including T790M mutation, while sparing wild-type EGFR [53, 54]. Dr. Jänne showed that in a group of 253 lung cancer patients with or without *EGFR*^*T790M*^ mutation receiving osimertinib at a dose of 20–240 mg/day, the median PFS is much longer in EGFR T790M-positive patients than in EGFR T790M-negative patients (9.6 months vs 2.8 months), suggesting that advanced NSCLC patients with the *EGFR*^*T790M*^ mutation were extraordinary sensitive to osimertinib (95% CI = 8.3–not reached) [55]. Different technology platforms have been used to assess the sensitivity and specificity of osimertinib to patients with *EGFR*^*T790M*^ mutation. Results showed that patients with *EGFR*^*T790M*^ mutation possess high sensitivity and specificity to osimertinib [56]. All observations suggest that patients with T790M mutation or resistance to EGFR TKI could be benefited from osimertinib.

#### CT16

CT16, targeting both EGFR and Notch2/3 receptors, not only inhibited signaling mediated by these receptors, but also showed a strong anti-stem cell effect both *in vitro* and *in vivo*. Data showed CT16 limits acquiring resistance to EGFR inhibitors and radiation in NSCLC cell line and patient-derived xenograft tumors. On the other hand, CT16 had a superior radio-sensitizing impact than other EGFR inhibitors [57]. These findings suggest that CT16 combining radiation may reveal clinical efficacy for patients with NSCLC.

### PI3KCA mutation and PI3Ks inhibitors

#### PI3KCA mutation

Phosphatidylinositol 3-kinases (PI3Ks) closely associates with cell proliferation, survival, adhesion and neoplasia [58]. The mutation of phosphatidylinositol 3-kinases catalytic alpha (PIK3CA) gene may play a role as an oncogene in human cancers [59], frequently occurring at exons 9 and 20 [60]. Mutant PIK3CA was found in approximately 4% NSCLC [60, 61].

### PI3Ks inhibitors

#### PX-866

PX-866 is an irreversible, pan-isoform inhibitor of PI3Ks. Nathan *et al.* have shown that PX-866 improved the antitumor activity of gefitinib in A549 NSCLC xenografts [62]. However, the main results were divergent in a randomized, phase II trial: 95 patients with advanced, recurrent, or metastatic NSCLC randomly assigned to docetaxel (75 mg/m^2^, intravenous) with (group A, 8 mg/day, orally) or without PX-866 (group B). The median PFS was 2 months in group A and 2.9 months in group B (*p* = 0.65); the ORR was 6% and 0% in group A and group B, respectively (*p* = 0.4). There was no difference in OS between the two groups (7.0 months vs 9.2 months, *p* = 0.9) [63]. Diarrhea (7%), vomiting (7%) and nausea (4%) occurred more in group A, with less number of grade 3 or higher adverse events found in both two groups [63]. The observations suggest that the efficacy of PX-866 is dependent on the treatment strategies for NSCLC.

#### Pictilisib (GDC-0941)

Pictilisib is an oral class I PI3Ks inhibitor that is being evaluated in phase II clinical trials for the treatment of breast and NSCLC [64]. Pictilisib has shown good safety and tolerability for NSCLC in the phase Ia/Ib study: Japanese patients with non-squamous NSCLC or advanced solid tumors received pictilisib in monotherapy (group A) or in combination with carboplatin-paclitaxel and bevacizumab (group B). The dose escalation of pictilisib was designed in this study for two stages: 140, 260, or 340 mg/day of pictilisib was administrated as monotherapy (stage 1) and 260 or 340 mg/day of pictilisib plus chemotherapy and bevacizumab was administrated (stage 2). In stage 1, only grade 3 maculopapular rash was found at the dose of 340 mg/day in group A, while an objective anti-tumor response was not observed. In stage 2, only grade 3 febrile neutropenia occurred at the dose of 260 mg/day or 340 mg/day in group B, while partial anti-tumor responses were observed in 3 out of 7 patients [65]. The efficacy of pictilisib for NSCLC is yet to be established in clinical trials.

#### Buparlisib (BKM120)

Buparlisib is a pan-PI3Ks inhibitor. Buparlisib mediated cell growth arrest and apoptosis of NSCLC through down-regulating the expression of Mcl-1 [66]. It has also been reported that buparlisib up-regulated the levels of auto phagosome-bound type II LC3 (LC3-II) protein, contributing to autophagy and disappearing of NSCLC [66]. However, in the phase II BASALT-1 study: 63 patients (30 squamous and 33 non-squamous) with activated PI3K pathway, metastatic, squamous or non-squamous NSCLC, who failed previous antineoplastic therapies (chemotherapy for squamous or systemic antineoplastic for non-squamous) received buparlisib (100 mg/day) for 12 weeks. The PFS rate was only 23.3% (95% CI = 9.9–42.3) and 20.0% (95% CI = 7.7–38.6) in the squamous and non-squamous groups, respectively [67], possibly due to overlapping signaling of treatments former and latter. Furthermore, both *in vitro* and *in vivo* studies show that buparlisib plus MEK162 (a MEK inhibitor) highly improve therapeutic efficacy against the growth of NSCLC cells [68]. Pre-clinical studies also showed that buparlisib combined with chloroquine inhibited lung cancer cells proliferation and induced apoptosis [69]. Combined therapies based on buparlisib hold promise for improving the clinical efficacy of patients with NSCLC.

#### mTOR inhibitors

Rapamycin binds two proteins (FK- 506 binding protein and FKBP-rapamycin-associated protein) as a potent immunosuppressive agent to inhibit mTORC1 [70]. Rapamycin has many derivatives including sirolimus, everolimus, ridaforolimus.

#### Sirolimus

Sirolimus is an oral mTOR inhibitor presenting unsatisfactory clinical efficacy. In a phase Ib trial, 39 NSCLC patients with EGFR mutation were randomly assigned to afatinib (40 mg/day) plus sirolimus (5 mg/day). The PFS was 6 months (33.3%). Adverse events (diarrhea: 94.9%, mucosal inflammation: 64.1%, asthenia: 53.8% and rash: 53.8%) were observed in all patients. Positive responses occurred in only 5 patients (12.8%) [71]. The results showed the efficacy of afatinib (40 mg/day) plus sirolimus was very much limited.

#### Everolimus

Everolimus is another novel inhibitor of mTOR pathway, yet demonstrating poor relatively clinical efficacy of combining with other drugs. In a phase II study, 28 patients with advanced stage NSCLC that previous had chemotherapy were treated with everolimus (5 mg/day) and docetaxel (60 mg/m^2^). The partial response occurred in 2 patients and stable disease was found in 15 patients (clinical benefit rate, 70%). The PFS was 6 months and OS was 9.6 months in everolimus plus docetaxel treatment. The results showed that everolimus plus docetaxel was tolerated well without satisfactory efficacy for patients with NSCLC [72]. Similarly, in another multicenter, open-label, phase II study, 133 patients with advanced NSCLC that previous had chemotherapy were randomly assigned to everolimus (5 mg/day) plus erlotinib (150 mg/day) and erlotinib alone. The disease control rate (DCR) at 3 months in everolimus plus erlotinib group was 39.4% and in erlotinib alone group was 28.4%. The median PFS was 2.9 months in everolimus plus erlotinib group and 2.0 months in erlotinib alone group. Grade 3/4 adverse events occurred in 72.7% and 32.3% of patients in everolimus plus erlotinib group and erlotinib alone group, respectively. Grade 3/4 stomatitis was only found in combination therapy (31.8%) [73]. These observations overall suggest that, everolimus plus erlotinib in combination cannot be a novel treatment strategy for individuals with NSCLC.

#### Ridaforolimus

Ridaforolimus is an investigational oral inhibitor targeting mTOR pathway. Clinical data showed that ridaforolimus is a good choice for patients with advanced NSCLC. In a randomized discontinuation phase II trial, 28 stage IIIB/IV NSCLC patients with KRAS mutation were treatment with ridaforolimus (40 mg/day) or placebo. The median PFS based on investigator assessment was obviously longer in ridaforolimus group (4 months) than placebo group (2 months, HR = 0.36, *p* = 0.013). The median OS was 18 months and 5 months in the ridaforolimus and placebo group, respectively (HR = 0.46, *p* = 0.09). More than 3 grade adverse events (fatigue: 10%, mucositis/stomatitis: 10%, pneumonia: 10%, diarrhea: 6%, dyspnea: 9%, and hyperglycemia: 6%) were observed in ridaforolimus group [74].

### ErbB2 amplification and ErbB2 inhibitors

#### ErbB2 amplification

Human epidermal growth factor receptor 2 (ErbB2, HER2), a member of the human EGFR (ErbB) family, is a receptor tyrosine kinase and encoded by the HER2 gene located on the long arm of chromosome 17 (17q21). ErbB2 is involved in activation of its downstream signaling pathways including PI3K/Akt as well as MEK/ERK, and necessary for cell proliferation and migration [75, 76]. ErbB2 amplification is found in 10–20% of NSCLC, while ErbB2 protein over-expression is observed in 2.4–38% of NSCLC [77–79].

### ErbB2 inhibitors

#### Neratinib (HKI-272)

Neratinib is an oral, irreversible dual EGFR/HER2 inhibitor. *In vitro*, neratinib could inhibit proliferation by inducing G1 arrest and apoptotic of lung cancer cells [80, 81]. However, clinical data showed that the efficacy of neratinib for NSCLC was poor and low efficacy of neratinib for NSCLC patients had been observed in a phase II trial: NSCLC patients were assigned to group A (EGFR mutation, *n* = 91), group B (wild-type EGFR, *n* = 48), and group C (TKI-naïve, *n* = 28). All of these patients were received neratinib daily. The ORR was 3% in group A and zero in both groups B and C. The most common toxicity was diarrhea. Obviously, 3 of 4 patients with *EGFR*^*G719X*^ mutation had a partial response [82]. Another phase I study also reported that neratinib plus temsirolimus treatment for NSCLC is associated with poor efficacy with side effects (diarrhea, nausea and stomatitis) [83]. Generally, further study will be needed for neratinib.

#### Lapatinib (GW572016)

Lapatinib is an oral, reversible, dual tyrosine kinase inhibitor of EGFR and HER2. The clinical efficacy of lapatinib monotherapy is uncertainty for NSCLC. In a randomized phase II study, patients with relapse or metastatic NSCLC were treated with lapatinib (1,500 mg/day or 500 mg/bid, orally). There were no complete or partial responses in the targeted population (*n* = 56): 25% had stable disease lasting 6 months. No responses were observed in patients with EGFR mutations (*n* = 3) and EGFR gene amplification (*n* = 5). 21% had stable disease lasting 6 months and 1.3% had partial response of patients in the nontargeted population (*n* = 75). Diarrhea, rash, fatigue, nausea, and anorexia were the most common adverse events [84]. The combination of lapatinib with other agents for treatment of NSCLC patients may be tested to improve the efficacy in the future.

#### VEGFR inhibitors

Vascular epidermal growth factor receptor (VEGFR) plays an important role in regulating of physiological angiogenesis that is closely associated with embryogenesis and tumor progress [85]. New blood vessel formation (angiogenesis) is required for the process of tumor growth and metastatic dissemination. Avascular tumors are severely restricted in their growth potential because of the lack of a blood supply [86]. Therefore, anti-angiogenesis is a valuable new approach to cancer therapy.

#### Bevacizumab

Bevacizumab, a monoclonal antibody against vascular endothelial growth factor (VEGF), benefits patients with NSCLC. It was applied as the first-line treatment of locally advanced, recurrent, unrespectable, or metastatic NSCLC in combination with chemotherapy (carboplatin and paclitaxel) that was approved by FDA in 2006 [87]. In a prospective cohort study, 102 patients with non-squamous NSCLC (44.1% of which carried EGFR mutation) received bevacizumab (15 mg/kg) with standard chemotherapy. The ORR was 44.1%, and the median PFS was 8.3 months in bevacizumab with standard chemotherapy (95% CI = 6.4–10.2). The median OS was 26.3 months (95% CI = 22.2–30.4). Grade 3-4 hypertension (30.4%) was observed in this study [88]. The results of this study showed that bev-containing chemotherapy was effective and safe for patients with non-squamous NSCLC. In another pilot phase II study, 39 patients with advanced NSCLC were randomly assigned to treatment with paclitaxel (80 mg/m^2^) and gemcitabine (300 mg/m^2^, weekly) for 3 weeks, plus bevacizumab (10 mg/kg, every two weekly). The median PFS was 8.5 months, the ORR was 56% and OS was 25.5 months. Myelosuppressive, gastrointestinal or neurologic events were not found in this study [89]. In addition, bevacizumab exhibited sufficient efficacy and safety for patients with NSCLC who failed first-line platinum-based chemotherapy: in a randomized phase III study, 297 Chinese patients were assigned to two groups: bevacizumab plus erlotinib and panitumumab (group A), or erlotinib plus placebo (group B). The median PFS in group A was much longer than in group B (4.6 months: 95% CI = 2.3–9.4 vs 1.9 months: 95% CI = 0.8–5.2, *p* = 0.003). The median OS was 10.4 months (95% CI = 7.5–13.1) and 8.9 months (95% CI = 3.3–10.9) in group A and group B (*p* = 0.031), respectively. Partial response in group A was 38%, significantly higher than in group B (15%; *p* = 0.014). In addition, diarrhea, fatigue and rash were occurred more in group B rather than group A [90]. These observations demonstrate that bevacizumab combination with other agents may have higher efficacy for patients with NSCLC.

#### Sorafenib

Sorafenib is an oral, multi-kinase inhibitor that was beneficent for NSCLC. In a multicenter phase II trial, 168 patients were randomly assigned to sorafenib (400 mg/day, bid) plus erlotinib (150 mg/day) or placebo plus erlotinib. The median PFS was 3.38 months and 1.94 months in sorafenib plus erlotinib and in placebo plus erlotinib, respectively (HR = 0.86, 95% CI = 0.60–1.22, *p* = 0.196). The disease control rate was 54% in sorafenib plus erlotinib and 38% in placebo plus erlotinib (*p* = 0.056) [91]. Similar clinical efficacy has been showed in another single-arm and multicenter phase II trial: 53 patients with advanced NSCLC were assigned to sorafenib (800 mg/day). The median PFS and median OS were 2.7 months and 6.7 months, respectively [92]. These results indicate that sorafenib monotherapy or combination with other agents may be promising for NSCLC treatment.

### Sunitinib

Sunitinib is an oral, multitargeted TKI targeting VEGFR, Kit, platelet-derived growth factor (PDGFR), FMS-like tyrosine kinase 3 (FLT3) and RET on tumor cells, tumor neovasculature and pericytes [93]. Clinical trials revealed that sunitinib had a certain extent efficacy to NSCLC with some side effects. In a phase III trial, 960 patients were randomly assigned to sunitinib (37.5 mg/day) plus erlotinib (150 mg/day) or placebo plus erlotinib. The median PFS was 3.6 months in sunitinib plus erlotinib versus 2.0 months in erlotinib alone (HR = 0.807; 95% CI = 0.695–0.937, *p* = 0.0023), and ORR was 10.6% versus 6.9% in sunitinib plus erlotinib and in erlotinib alone (*p* = 0.0471), respectively. The median OS was 9.0 months and 8.5 months in sunitinib plus erlotinib and in erlotinib alone, respectively (HR = 0.922; 95% CI = 0.797–1.067, *p* = 0.1388). However, rash/dermatitis, diarrhea and asthenia/fatigue were more frequently observed in the sunitinib plus erlotinib group [94]. The same situation happened in another randomized, double-blind, placebo-controlled phase III study: 210 patients with stage IIIB/IV NSCLC who experiencing first-line chemotherapy were randomized to receive sunitinib (37.5 mg/day) or placebo. The median PFS was 4.3 months in sunitinib and 2.6 months in placebo (HR = 0.62, 95% CI = 0.47–0.82, *p* = 0.0006). The median OS was 11.7 months in sunitinib versus 12.1 months in placebo (HR = 0.98, 95% CI = 0.73–1.31, *p* = 0.89). Serious effects including fatigue (25%), thrombocytopenia (12%), hypertension (12%) and rash (11%) were occurred in sunitinib [95]. These results indicate that sunitinib in combination or alone is beneficial for advanced NSCLC.

### KRAS mutation and KRAS inhibitors

#### KRAS mutation

In NSCLC, and especially in lung adenocarcinomas, signaling of kirsten human rat sarcoma protein (KRAS), a main downstream signaling molecule of EGFR pathway, is frequently influenced by its own somatic mutations [96, 97]. Mutations of EGFR and KRAS are regarded as primary genetic “driver” leading to cancer [98]. KRAS is frequently activated in NSCLC, and KRAS gene mutations were found in 20% of NSCLC. Most KRAS mutations reported in NSCLC were found at codon 12 [97]. Although KRAS mutations were identified in lung cancer, there have been very few trials addressing NSCLC patients with KRAS mutations, leading to no positive therapies [99]. However, there is a dire need for therapies specifically for patients with KRAS mutant NSCLC.

### KRAS inhibitor

#### Antroquinonol

Antroquinonol is a small anticancer molecule isolated from Antrodia camphorate which shows highly anticancer activity [100, 101]. Antroquinonol inhibited Ras and Ras-related small GTP-binding protein functions by binding directly to farnesyltransferase and geranylgeranyl transferase-I (the key enzymes associated with activation of Ras-related proteins). Furthermore, antroquinonol induced autophagy by increasing the level of light chain 3 B- II (LC3B-II) and the auto phagosome-associated LC3 [102]. In an open-label, dose escalation, pharmacokinetic (PK) study, 13 patients with metastatic adenocarcinoma NSCLC were treated with antroquinonol (escalating doses of 50–600 mg/day). No dose-limiting toxicities was seen in any patient at any dose level. The mean elimination half-life is between 1.30 and 4.33 h, which associates with the treatment dose. A mild toxicity profile was observed at all dose levels of antroquinonol. Diarrhea, vomiting and nausea occurred in this study. The best tumor response was stable disease in 3 patients [103]. This study showed that antroquinonol was good, safe and tolerated for NSCLC. The efficacy of antroquinonol in clinical trials have still to be evaluated.

### c-MET amplification and c-MET inhibitors

#### c-MET amplification

Cellular-mesenchymal to epithelial transition factor (c-MET) is a plasma membrane tyrosine kinase activated by auto-phosphorylation after ligand binding. The c-MET was significantly associated with tumor growth in NSCLC [104]. Additionally, a recent study has shown that MET amplifications are present in 9 of 43 patients with acquired resistance to EGFR TKI (gefitinib and erlotinib) [105]. MET amplification was also observed in 4 of 18 lung cancer patients with gefitinib or erlotinib-resistance [106]. These data suggest that MET amplification may play an important role in promoting drug resistance. The c-MET receptor is an attractive potential target for novel therapeutic inhibition in human cancers.

### c-MET inhibitors

#### Tivantinib

Tivantinib, an oral MET receptor TKI, exhibited high anticancer activity in early clinical studies and preclinical studies when combined with erlotinib. In a phase III multinational, randomized, double-blind, placebo-controlled study, 1,048 patients with advanced non-squamous NSCLC were randomly assigned to receive erlotinib (150 mg/day) plus tivantinib (720 mg/day) or erlotinib plus placebo. The median PFS was 3.6 months in erlotinib plus tivantinib and 1.9 months in erlotinib alone (HR = 0.74, 95% CI = 0.62–0.89, *p* < 0.001). The median OS was 8.5 months versus 7.8 months in erlotinib plus tivantinib and in erlotinib alone, respectively (HR = 0.98, 95% CI = 0.84–1.15, *p* = 0.81). Diarrhea (34.6% vs 41.0%), rash (33.1% vs 37.3%), neutropenia (grade 3 to 4; 8.5% vs 0.8%) and asthenia or fatigue (43.5% vs 38.1%) were seen in both erlotinib plus tivantinib and in erlotinib group [107].

#### Onartuzumab (MP470)

Onartuzumab is a monovalent monoclonal antibody targeting MET. Clinical data showed that onartuzumab is a good choice for MET-positive patients with NSCLC. In a randomized phase II trial, patients with recurrent NSCLC were randomly assigned to receive onartuzumab plus erlotinib or placebo plus erlotinib [108]. No definite conclusion has been derived in PFS (2.2 months vs 2.6 months; HR = 1.09, *p* = 0.69) or OS (8.9 months vs 7.4 months; HR = 0.80, *p* = 0.34) in the intent-to-treat (ITT) population (*n* = 137). However, both PFS (2.9 months vs 1.5 months; HR = 0.53, *p* = 0.04) and OS (12.6 months vs 3.8 months; HR = 0.37, *p* = 0.002) were improved for the 66 MET-positive (immunohistochemistry diagnostic positive) patients that were treated with erlotinib plus onartuzumab. Conversely, worse clinical outcomes were occurred in 62 MET-negative (immunohistochemistry diagnostic negative) patients that received onartuzumab plus erlotinib (PFS: 1.4 months vs 2.7 months; HR = 1.82, *p* = 0.05; OS: 8.1 months vs 15.3 months; HR = 1.78, *p* = 0.16). Incidence of peripheral edema was observed in onartuzumab-treated patients with increased trend [108]. Another phase II study showed the same trend upon onartuzumab treatment for NSCLC: 109 patients with advanced squamous NSCLC were randomly assigned to treat with onartuzumab plus chemotherapy (paclitaxel plus carboplatin/cisplatin) or placebo plus chemotherapy. Similar results were obtained between onartuzumab plus chemotherapy and placebo plus chemotherapy in term of the risk of disease progression or death in ITT (stratified HR = 0.95, 95% CI = 0.63–1.43) and MET immunohistochemistry diagnostic positive (IHC^+^) populations (unstratified HR = 1.27; 95% CI = 0.69–2.32). The grade 3 to 5 adverse events (neutropenia and pulmonary embolism) were observed 14.8% and 5.8% in the onartuzumab plus chemotherapy and in the placebo plus chemotherapy, respectively [109].

#### Cabozantinib (XL-184)

Cabozantinib is a multi-kinase inhibitor targeting MET and VEGFR2. In a phase II study, patients with NSCLC were randomized to treat with cabozantinib alone (100 mg/day) or cabozantinib (100 mg/day) plus erlotinib (50 mg/day). The ORR was 6.7% (90% CI = 0.3–27.9) in the cabozantinib alone (*n* = 15) with no responses in cabozantinib plus erlotinib (*n* = 13) [110]. There was no evidence to show that combination of cabozantinib plus erlotinib significantly sensitizes cabozantinib in patients with NSCLC.

#### Su11274

Su11274, the c-MET-specific TKI, in combination with EGFR TKIs were used to treat NSCLC cells. One of recent studies showed that su11274 potentiated the ability of afatinib against *EGFR*^*T790M*^ H1975 cells [111]. This study has provided evidences that combined inhibition of c-MET and EGFR would be a good clinical treatment strategy for NSCLC patients.

#### PHA-665752

PHA-665752 is a small molecule, inhibitor of c-MET kinase. PHA-665752 dramatically suppresses tumor growth, as well as angiogenesis, in mouse NCI-H441 xenografts model [112]. The study suggested that PHA-665752 may be a novel inhibitor of lung cancer. Additional clinical trials of PHA-665752 for NSCLC are required in the future.

### ALK fusion and ALK inhibitors

#### ALK fusion

Anaplastic lymphoma kinase (ALK), a tyrosine kinase receptor, was found in anaplastic large-cell lymphoma (ALCL) cell lines in 1994 as a member of the insulin receptor superfamily which was closely associated with both solid and hematological tumors [113]. ALK gene rearrangements have described in NSCLC. The echinoderm microtubule-associated protein-like 4 gene and the anaplastic lymphoma kinase gene (EML4-ALK) fusion oncogene was identified as a novel effective target in NSCLC in 2007 which has been detected in 5% NSCLC patients [114–116]. EML4-ALK fusions have been manifested to be mutually exclusive with EGFR or KRAS mutation found in patients without smoking history [117, 118]. However, drug resistance has been identified for crizotinib which was contributed by secondary mutations and therefore activation of either a bypass signal pathway (KIT or EGFR), or the ALK gene itself (such as F1174L, L1196M, C1156Y, G1269A, S1206Y, and G1202R), or gene amplification [119, 120].

### ALK inhibitors

#### Crizotinib

Crizotinib, as a first generation ALK tyrosine kinase inhibitor, has been approved by US FDA in 2014 [121]. Crizotinib showed highly efficacy in NSCLC patient with ALK rearrangements, targeting ALK, MET, ROS1 [122]. In a single-arm, open-label, phase I–II study, NSCLC patients harboring ALK rearrangements (phase I, *n* = 24; phase II, *n* = 46) were given crizotinib (phase I, 20–300 mg/bid; phase II, 300 mg/bid). In Phase I, no adverse events of grade 4 were founded up to the highest dose. In Phase II, the objective response rate was 93.5% (*n* = 43, 95% CI = 82.1–98.6). Adverse events of grade 3 were noted in 12 of 46 patients, and no grade 4 adverse events or deaths were recorded [123]. In another phase 3 trial, 343 patients with advanced ALK-positive non-squamous NSCLC were randomly assigned to receive crizotinib (250 mg/bid) or chemotherapy (pemetrexed or plus either cisplatin, or carboplatin). The median PFS was significantly longer in crizotinib group than in chemotherapy group (10.9 months vs. 7.0 months, HR = 0.45, 95% CI = 0.35–0.60; *p* < 0.001). Patients in crizotinib group showed highly reduction in lung cancer symptoms and better improvement in quality of life [124].

#### Alectinib

Alectinib, as a second-generation highly selective small-molecule ALK inhibitors, shows great efficacy for overcoming crizotinib resistance in NSCLC patients with ALK rearrangements [125]. In a phase II study, 87 patients had received prior crizotinib therapy were randomly assigned to receive alectinib. The ORR was 48% (95% CI = 0.36–0.60). Grade 1 or 2 adverse events (peripheral edema, myalgia, fatigue and constipation) were observed in this study [126]. Alectinib also showed effective in the 3-year follow-up of phase II: patients with ALK positive NSCLC received alectinib (300 mg//bid). The 3-year PFS rate was 62% (95% CI = 45–75) and OS rate was 78% [127].

#### Ceritinib

Ceritinib is another ALK inhibitor which has been approved by FDA in 2014. It shows great clinical efficacy against crizotinib-resistant ALK-positive tumors. In a Multicenter Phase II Study, 140 patients with advanced ALK-rearranged NSCLC who had been previously treated with crizotinib were randomly assigned to given ceritinib. The median PFS was 5.7 months (95% CI = 5.4–7.6), the overall response rate was 38.6% (95% CI = 0.30–0.47) and disease control rate was 77.1% (95% CI = 0.69–0.84) [128]. In another Open-Label, Multicenter, Phase II Study, 32 patients with advanced ALK-positive NSCLC were randomly assigned to receive ceritinib (750 mg/day). The median PFS and median OS were 9.3 months (95% CI = 0–22) and 24 months (95% CI = 5–43), respectively. The ORR was 62% (95% CI = 0.45–0.77) and the duration of response was 21.0 months (95% CI = 17–25); DCR was 81% (95% CI= 0.65–0.91) [129].

### BRAF mutation and BRAF inhibitor

#### BRAF mutation

vRaf murine sarcoma viral oncogene homolog B (BRAF), as a proto-oncogene, encodes a serine/threonine protein kinase that promotes cell survival and proliferation [130]. BRAF mutations have been identified in a wide range of malignancies including 10% of colorectal carcinoma [131], and 50% of malignant melanomas [132]. The BRAF Val600Glu (V600E) mutation were commonly seen in BRAF mutations that had been mainly found approximately 90% of melanoma [133]. The frequency of BRAF V600E mutation in NSCLC is about 1%–3% [134].

### BRAF inhibitor

#### Dabrafenib (GSK2118436)

Dabrafenib, as an oral selective BRAF inhibitor [135]. Dabrafenib has shown activity in patients with advanced NSCLC harboring the BRAF V600E mutation. In a phase II study, 84 advanced NSCLC patients with BRAF V600E mutation were treated with dabrafenib (150 mg/bid). The median PFS of this study was 5.5 months (95% CI = 3.4–7.3) and the ORR was 33% (95% CI = 0.23–0.45). The median OS was 12.7 months (95% CI = 7.3–16.9) [136]. However, combining dabrafenib with trametinib (a MEK inhibitor) may reveal a more effective treatment strategy. In an open-label, phase II trial, 36 advanced NSCLC patients with BRAF V600E mutation patients were randomly assigned to receive dabrafenib plus trametinib. The median OS was 24.6 months (95% CI = 12.3–not estimable) and the ORR was 64% [137].

## CONCLUSIONS AND PERSPECTIVES

Recently, a series of achievements have been made in NSCLC treatment with the better understanding of the molecular biology of NSCLC. Molecular targeted therapy based on targeting specificity can improve treatment safety and efficiency for NSCLC patients (Tables 1 and 2), although there are still challenges in development and application of targeted drugs. First of all, the efficacy of targeted drugs is yet to be improved alone with the potential side effects of the drugs; secondly, effective drug combinations hold promise and await further development. Furthermore, acquired resistance needs to be dealt with great efforts due to the constant occurrence after targeted therapies. With concerted efforts in these fields, the ultimate goal of achieving effective and harmless NSCLC therapy may be achievable.

**Table 1:** Targeted therapies for NSCLC

Inhibitor(s)	Target(s)	Clinical trial (Phase)	Reference(s)
Gefitinib	EGFR mutation: exon 19 deletions or exon 21 (*EGFR* ^*L858R*^) mutations	III	[34]
Erlotinib	EGFR mutation: exon 19 deletions or exon 21 (*EGFR* ^*L858R*^) mutations	II	[40]
Icotinib	EGFR mutation: exon 19 deletions or exon 21 (*EGFR* ^*L858R*^) mutations	III	[41]
Afatinib	WT EGFR and EGFR mutation: exon 19 deletions or exon 21 (*EGFR* ^*L858R*^) mutations	III	[44, 45]
Dacomitinib	WT EGFR and EGFR mutation: exon 19 deletions or exon 21 (*EGFR* ^*L858R*^) mutations	II, III	[49, 50]
Osimertinib	*EGFR* ^*T790M*^ mutation	II, III	[56]
CT16	EGFR, Notch2/3	-	-
PX-866	PI3K	II	[63]
Pictilisib	PI3K	I	[65]
Buparlisib	PI3K	II	[67]
Sirolimus	mTOR	I	[71]
Everolimus	mTOR	II	[72, 73]
Ridaforolimus	mTOR	II	[74]
Neratinib	ErbB2 amplification: the long arm of chromosome 17 (17q21)	I, II	[82, 83]
Lapatinib	EGFR, HER2	II	[84]
Bevacizumab	VEGFR	II, III	[89, 90]
sorafenib	VEGFR	II	[91, 92]
sunitinib	VEGFR, KIT, PDGFR, FLT3, RET	III	[94, 95]
Antroquinonol	KRAS mutation	I	[103]
Tivantinib	c-MET amplification	III	[107]
Onartuzumab	c-MET amplification	II	[108, 109]
Cabozantinib	c-MET, VEGFR	II	[110]
Su112749	c-MET amplification	-	-
PHA-665752	c-MET amplification	-	-
Crizotinib	ALK fusion	II, III	[123, 124]
Alectinib	ALK fusion	II	[126, 127]
Ceritinib	ALK fusion	II	[128, 129]
Dabrafenib	BRAF V600E	II	[136, 137]

Molecular targets, corresponding inhibitors and clinical trials are included.

**Table 2:** Results of clinical trails

Inhibitor	Subjects (*N*)	PFS			OS			ORR		
		Time (month)	*p*-value	95% CI	Time (month)	*p*-value	95% CI	Rate (%)	*p*-value	95% CI
Gefitinib	261	10.9	0.001	0.4–0.8	-	-	-	67.0	0.0004	1.6–5.5
Erlotinib	154	16.0	0.002	13.9–18.1	-	-	-	-	-	-
Icotinib	400	4.6	0.1	3.5–6.3	13.3	-	11.1–16.2	62.1	0.5	0.5–3.8
Afatinib	910	11.0	<0.0001	9.7–13.7	22.1	0.8	20.0-not estimable	66.9	<0.0001	4.3–12.2
Dacomitinib	121	14.6	0.2	9.0–18.2	26.6	-	20.1–29.0	62.1	-	0.5–0.7
Osimertinib	ongoing	ongoing	ongoing	ongoing	ongoing	ongoing	ongoing	ongoing	ongoing	ongoing
PX-866	95	2.0	0.7	-	7.0	0.9	-	6.0	0.4	-
Buparlisib	63	3.0	-	9.9–42.3	8.0	-	6.0–10.1	3.3	-	0.1–7.2
Sirolimus	39	3.4	-	1.8–6.3	-	-	-	-	-	-
Everolimus	133	2.9	0.2	2.4–3.9	9.1	-	7.5–11.1	-	-	-
Ridaforolimus	28	4.0	0.01	-	18.0	0.09	-	-	-	-
Neratinib	167	15.3	-	14.7–15.9	-	-	-	-	-	-
Lapatinib	75	3.7	-	-	14.5	-	-	-	-	-
Bevacizumab	297	4.6	0.003	2.3–9.4	10.4	0.03	7.5–13.1	38.0	0.01	-
sorafenib	168	3.4	0.2	0.6–1.2	6.7	8.0	0.19	8.0	0.6	-
sunitinib	960	3.6	0.002	0.7–0.9	9.0	0.2	0.8–1.1	10.6	0.05	-
Tivantinib	1048	3.6	0.001	0.6–0.9	8.5	0.8	0.8–1.2	-	-	-
Onartuzumab	137	2.9	0.04	-	12.6	0.002	-	5.8	-	-
Cabozantinib	65	3.9	-	1.5–7.3	-	-	-	6.7	-	0.3–27.9
Crizotinib	343	10.9	<0.001	0.4–0.6	17.4	0.4	0.5–1.3	74.0	<0.001	-
Alectinib	87	8.1	-	6.2–11.6	12.0	-	61.0–88.0	48.0	-	0.4–0.6
Ceritinib	140	5.7	-	5.4–7.6	14.9	-	13.5–not estimable	38.6	-	0.3–0.5
Dabrafenib	84	5.5	-	3.4–7.3	12.7	-	7.3–16.9	33.0	-	0.2–0.5

The results of molecular target inhibitors of NSCLC are summarized including PFS, OS and ORR.

## Abbreviations

NSCLC: Non-small cell lung cancer
EGFR: Epidermal growth factor receptor
PI3K: Phosphatidylinositol 3-kinases
mTOR: Mechanistic target of rapamycin
ErbB2: Epidermal growth factor receptor 2
VEGF: Vascular endothelial growth factor
VEGFR: Vascular endothelial growth factor receptor
RAS: Human rat sarcoma protein
KRAS: Kirsten human rat sarcoma protein
c-MET: Mesenchymal-epithelial transition factor or hepatocyte growth factor receptor
PFS: Progression free survival
OS: Median overall survival
RFS: Recurrence-free survival
HR: Hazard Ratio
CI: Confidence interval
TKI: Tyrosine kinase inhibitor
FDA: Food and Drug Administration
DoR: Duration of response
IPASS: Iressa Pan-ASia Study
ITT: Intent-to-treat
OR: Odds ratio
ORR: Objective response rate
US: United States
CFDA: China Food and Drug Administration
MEK: Mitogen-activated protein kinase
DCR: Disease control rate
MAPK10: Mitogen activated protein kinase 10
PIM-1: Polymer of intrinsic microporosity
DHODH: Dihydroorotate dehydrogenase
ERBB-4: Epidermal growth factor receptor 4
HSD17B1: 17β Hydroxysteroid dehydrogenase type 1
CHK2: Checkpoint kinase-2
CHK1: Checkpoint kinase-1
Bcl-2: B-cell lymphoma-2
Bcl-xl: B-cell lymphoma-xl
Mcl-1: Myeloid cell leukemia
LC3: Light chain 3
ERK: Extracellular Signal-regulated Kinase
RAF: Rapidly accelerated fibrosarcoma
JAK: Janus kinase
Shc: Generic shell script compiler
Src: Steroid receptor coactivator
GSK-3: Glycogen synthase kinase 3
Bmi: Body mass index
STAT3: Signal transducer and activator of transcription 3
DNA: Deoxyribonucleic acid
RNA: Ribonucleic acid
PDGFR: Platelet-derived growth factor
FLT3: Fms-like tyrosine kinase 3
ALK: anaplastic lymphoma kinase
BRAF: V-Raf murine sarcoma viral oncogene homolog B
